# Mutant Analysis Reveals Allosteric Regulation of ClpB Disaggregase

**DOI:** 10.3389/fmolb.2017.00006

**Published:** 2017-02-22

**Authors:** Kamila B. Franke, Bernd Bukau, Axel Mogk

**Affiliations:** Center for Molecular Biology of the Heidelberg University, German Cancer Research CenterHeidelberg, Germany

**Keywords:** AAA+ protein, ClpB, Hsp104, protein disaggregation, arginine finger

## Abstract

The members of the hexameric AAA+ disaggregase of *E. coli* and *S. cerevisiae*, ClpB, and Hsp104, cooperate with the Hsp70 chaperone system in the solubilization of aggregated proteins. Aggregate solubilization relies on a substrate threading activity of ClpB/Hsp104 fueled by ATP hydrolysis in both ATPase rings (AAA-1, AAA-2). ClpB/Hsp104 ATPase activity is controlled by the M-domains, which associate to the AAA-1 ring to downregulate ATP hydrolysis. Keeping M-domains displaced from the AAA-1 ring by association with Hsp70 increases ATPase activity due to enhanced communication between protomers. This communication involves conserved arginine fingers. The control of ClpB/Hsp104 activity is crucial, as hyperactive mutants with permanently dissociated M-domains exhibit cellular toxicity. Here, we analyzed AAA-1 inter-ring communication in relation to the M-domain mediated ATPase regulation, by subjecting a conserved residue of the AAA-1 domain subunit interface of ClpB (A328) to mutational analysis. While all A328X mutants have reduced disaggregation activities, their ATPase activities strongly differed. ClpB-A328I/L mutants have reduced ATPase activity and when combined with the hyperactive ClpB-K476C M-domain mutation, suppress cellular toxicity. This underlines that ClpB ATPase activation by M-domain dissociation relies on increased subunit communication. The ClpB-A328V mutant in contrast has very high ATPase activity and exhibits cellular toxicity on its own, qualifying it as novel hyperactive ClpB mutant. ClpB-A328V hyperactivity is however, different from that of M-domain mutants as M-domains stay associated with the AAA-1 ring. The high ATPase activity of ClpB-A328V primarily relies on the AAA-2 ring and correlates with distinct conformational changes in the AAA-2 catalytic site. These findings characterize the subunit interface residue A328 as crucial regulatory element to control ATP hydrolysis in both AAA rings.

## Introduction

AAA+ proteins constitute a protein superfamily sharing the ability to convert the chemical energy derived from ATP hydrolysis into mechanical work. AAA+ proteins share the AAA domain, which is defined by a region of ~230 amino acids in length, comprising conserved Walker A and Walker B motifs for nucleotide binding and hydrolysis. The AAA domain also drives protein oligomerization, frequently into hexameric ring-like structures with a central pore. The catalytic active site is located at the subunit interface of AAA domains involving conserved elements from both subunits (Miller and Enemark, 2016). AAA+ proteins differ in the number of AAA domains (one or two) per protomer and the presence of extra domains, which provide functional specificity by controlling substrate interactions.

Many AAA+ proteins function as ATP fueled unfolding machineries, causing disassembly of substrate complexes or coupling substrate unfolding to degradation via associated peptidases (Sauer and Baker, 2011). Substrate unfolding by AAA+ proteins is typically mediated by pulling at a bound substrate stretch leading to substrate threading through the central pore. This threading activity is executed by pore-located aromatic residues that are located on mobile loops, which move downwards the central channel in a nucleotide-controlled manner (Yamada-Inagawa et al., 2003; Schlieker et al., 2004; Zolkiewski, 2006).

How ATP hydrolysis is orchestrated and linked to the formation of a mechanical force is key to understand AAA+ protein function. The regulation of ATPase activity is complex. AAA+ proteins form asymmetric assemblies as not all AAA domains bind nucleotide at the same time. For various family members including ClpX, PAN, and ClpB it was shown that only four out of six nucleotide binding sites are occupied (Hersch et al., 2005; Glynn et al., 2009; Smith et al., 2011; Carroni et al., 2014). The individual AAA domains can in principle work independently and ATP hydrolysis can proceed in a probabilistic manner (Martin et al., 2005). However, coordination of ATP hydrolysis leads to power strokes with higher strengths that are linked to more efficient substrate threading (Sen et al., 2013).

The position of ATPase active sites at the interface of two neighboring AAA subunits offers a pathway for allosteric signal transmission and subunit coupling. Conserved arginine fingers located at the subunit interface contact the γ-phosphate of ATP bound in the neighboring subunit. Arginine fingers function as essential trans-acting elements in ATP hydrolysis and provide a structural framework to sense nucleotide states and to transmit this information across the AAA ring in an allosteric fashion (Karata et al., 1999; Wang et al., 2005; Zeymer et al., 2014b).

Hsp100 protein disaggregases (*Escherichia coli* ClpB, *Saccharomyces cerevisiae* Hsp104) harbor two AAA domains (AAA-1, AAA-2) and solubilize aggregated proteins in concert with a cognate Hsp70 chaperone system (Aguado et al., 2015b; Mogk et al., 2015). Hsp70-mediated recruitment of ClpB/Hsp104 to protein aggregates is coupled to ATPase activation (Seyffer et al., 2012; Lee et al., 2013; Rosenzweig et al., 2013). Hsp70 interaction and ATPase control are directly linked via the specific ClpB/Hsp104 M-domain. The M-domain forms a coiled-coil structure, which is composed of four helices forming two wings termed motif1 and motif2 (Lee et al., 2003). M-domain motif2 exists in two structural states. It is either in close contact with AAA-1 or dissociates from the AAA-1 ring, enabling its binding to Hsp70 (Oguchi et al., 2012; Carroni et al., 2014). The interaction between M-domain motif2 and AAA-1 downregulates ClpB/Hsp104 ATPase activity (Oguchi et al., 2012; Lee et al., 2013; Lipinska et al., 2013). This qualifies the AAA-1 ring of ClpB/Hsp104 as a main regulatory site, while the AAA-2 ring is suggested to represent the major ATPase motor for substrate threading (Mogk et al., 2015). M-domain mutants disrupting AAA-1/M-domain interaction exhibit high ATPase activities in presence of substrate, leading to increased unfolding power and disaggregation activities (Oguchi et al., 2012; Lipinska et al., 2013; Jackrel et al., 2014). Hyperactive M-domain mutants, however, exhibit temperature-dependent cellular toxicity rationalizing tight control of ClpB ATPase activity (Schirmer et al., 2004; Oguchi et al., 2012; Lipinska et al., 2013). The cellular targets of hyperactive M-domain mutants are largely unknown. Hyperactive ClpB/Hsp104 might act on endogenous proteins exposing a specific recognition tag for ClpB/Hsp104 interaction, leading to unfolding of the native protein. Hyperactive ClpB/Hsp104 could also interfere with the *de novo* folding of nascent polypeptides and the secretion of secretory proteins.

How the M-domain docking state signals to the ATPase center and which step in the ATPase cycle is modulated is currently unknown. Mixing experiments of ClpB/Hsp104 wild type and ATPase deficient subunits suggest that M-domain dissociation increases AAA subunit cooperation leading to high ATP turnover rates upon additional substrate binding (Seyffer et al., 2012; Lee et al., 2013; Aguado et al., 2015a; Kummer et al., 2016). Such allosteric control might involve the conserved arginine fingers of both ClpB/Hsp104 AAA domains (*E. coli* ClpB R331/R332 (AAA-1) and R756 (AAA-2). Arginine fingers are essential for ClpB/Hsp104 disaggregation activity (Mogk et al., 2003; Yamasaki et al., 2011; Biter et al., 2012). The arginine fingers are crucial for ATP hydrolysis in the respective AAA ring but also act as trans-acting elements, as they affect ATP hydrolysis in the second AAA ring as well (Mogk et al., 2003; Werbeck et al., 2011; Yamasaki et al., 2011; Biter et al., 2012). Arginine fingers thereby control ATPase regulatory circuits in both, cis and trans.

Here we analyzed the interplay between ClpB intersubunit communication within the first AAA domain and M-domain mediated ATPase control. We analyzed the effects of mutational alterations of a conserved subunit interface residue located close to the conserved arginine fingers of the first AAA domain. We show that small structural alterations at this position have profound and distinct effects on ATPase control, causing either strong reduction or increase of total ATPase activity. Affecting AAA-1 intersubunit signaling can overrule ATPase deregulation by ClpB M-domain mutants, suppressing hyperstimulation of ATPase activity and cellular toxicity. Together our findings confirm and extend our molecular understanding of ClpB interring communication in controlling ATPase and disaggregation activities.

## Materials and methods

### Strains, plasmids, and proteins

*E. coli* strains used were derivatives of MC4100. ClpB was amplified by PCR and inserted into pDS56 and verified by sequencing. Mutant derivatives of *clpB* were generated by PCR mutagenesis and standard cloning techniques in pDS56 and were verified by sequencing. ClpB was purified after overproduction from *E. coli* Δ*clpB::kan* cells. ClpB wild type and mutant variants were purified using Ni-IDA (Macherey-Nagel) and size exclusion chromatography (Superdex S200, Amersham) following standard protocols. Purifications of DnaK, DnaJ, GrpE, Luciferase, and Casein-YFP were performed as described previously (Haslberger et al., 2008; Oguchi et al., 2012; Seyffer et al., 2012). Pyruvate kinase of rabbit muscle and Malate Dehydrogenase of pig heart muscle were purchased from Sigma. Protein concentrations were determined with the Bio-Rad Bradford assay.

### Biochemical assays

#### Disaggregation assays

ClpB disaggregation activities were determined by following the disaggregation of heat-aggregated Malate Dehydrogenase (0.5 μM, 30 min at 47°C) and 0.05 μM urea-denatured firefly Luciferase at 25°C as described (Oguchi et al., 2012; Kummer et al., 2016). Chaperones were used at the following concentrations: 1 μM ClpB (wild type or derivatives), *E. coli* Hsp70 system: 1 μM DnaK, 0.2 μM DnaJ, 0.1 μM GrpE. Disaggregation reactions were performed in Reaction Buffer (50 mM Tris pH 7.5, 150 mM KCl, 20 mM MgCl_2_, 2 mM DTT) containing an ATP Regenerating System (2 mM ATP, 3 mM phosphoenolpyruvate, 20 ng/μl Pyruvate Kinase). Luciferase activities were determined with a Lumat LB 9,507 (Berthold Technologies) MDH disaggregation was monitored by turbidity measurement at an excitation and emission wavelength of 600 nm (PerkinElmer LS50B spectrofluorimeter).

Luciferase refolding rates and MDH disaggregation rates were calculated from the linear increase in Luciferase activities and linear decrease in MDH aggregate turbidity

#### ATPase assay

ATPase activities of ClpB (0.5 μM) was determined in Reaction buffer in absence or presence of substrate (10 μM casein) using a NADH-coupled colorimetric assay (Sigma) by measuring the decrease of NADH absorption at 340 nm in a BMG Labtech FLUOstar Omega plate reader. Minor differences in ATPase activities determined for ClpB wt and mutants (**Figures 3**, **6**) are caused by analysis of different protein purification batches.

#### Nucleotide binding

To determine the affinity of ClpB (wt and derivatives) for the fluorescent nucleotide analog mantADP equilibrium titrations of 1,25 μM mantADP with different ClpB concentrations were performed at 30°C using a FP 6500 JASCO Spectrometer (Excitation: 360 nm/Emission: 400–500 nm). The affinity for mantADP can be determined using the following equation:

$$\begin{array}{l} \text{F }=\;{\text{F}}_{0}\\ \;+\left({\text{F}}_{\text{max}}-{\text{F}}_{0}\right)\frac{\frac{{\left[\text{E}\right]}_{0}+{\left[\text{L}\right]}_{0}+{\text{K}}_{\text{d}}}{2}-\sqrt{\frac{{\left({\left[\text{E}\right]}_{0}+{\left[\text{L}\right]}_{0}+{\text{K}}_{\text{d}}\right)}^{2}}{4}-{\left[\text{E}\right]}_{0}{\left[\text{L}\right]}_{0}}}{{\left[\text{L}\right]}_{0}} \end{array}$$

with F: observed fluorescence; F_0_: fluorescence of fluorophor (mantADP); F_max_: maximum fluorescence observed; [E]_0_: total concentration of ClpB (μM); [L]_0_: total concentration of fluorophor (μM); K_d_: dissociation constant of the complex (μM).

To determine the affinity of ClpB wt and derivatives for ADP and ATPγS competition titrations were performed. 1,25 μM mantADP were initially mixed with 1,25 μM ClpB and pre incubated for 5 min at 30°C. Subsequently this complex was titrated with solutions of ADP and ATPγS and mantADP fluorescence as determined as described above. The affinities for the unlabeled nucleotides were determined using the following equations:

$$\begin{array}{l} \text{F }=\;\left[{\text{F}}_{0}\frac{{\text{K}}_{\text{i},\text{ app}}}{\left[\text{ATP}\right]+{\text{K}}_{\text{i},\text{app}}}\right]+\left[{\text{F}}_{1}\frac{\left[\text{ATP}\right]}{\left[\text{ATP}\right]+{\text{K}}_{\text{i},\text{app}}}\right] \end{array}$$

with F: observed fluorescence; F_0_: starting fluorescence without nucleotide; F_1_: maximum fluorescence observed; [ATP]: concentration of ADP/ATPγS (μM); K_i,app_: apparent dissociation constant.

$$\begin{array}{l} {\text{K}}_{\text{i},\text{app}}\;={\text{K}}_{\text{i}}\left(1+\frac{\left[\text{mantADP}\right]}{{\text{K}}_{\text{d}}\left(\text{mantADP}\right)}\right) \end{array}$$

with K_i,app_: apparent dissociation constant; K_i_: dissociation constant; [mantADP]: mantADP concentration (μM); K_d_ (mantADP): dissociation constant of mantADP.

#### Unfolding assays

Unfolding and degradation of Casein-YFP (0.25 μM) was determined in Reaction buffer in presence of 4 μM BAP (wt or derivatives) and 6 μM ClpP and an ATP regenerating system. YFP fluorescence was followed on a Perkin-Elmer LS50B spectrofluorimeter (excitation wavelength 488 nm, emission wavelength 527 nm).

#### Glutaraldehyde crosslinking

All tested ClpB variants were dialyzed against Reaction Buffer B (50 mM HEPES ph7.5, 25 mM KCl, 5 mM MgCl_2_, 2 mM DTT). One micromolar ClpB was incubated at 25°C in presence of ATPγS for 5 min. Crosslinking reactions were started by addition of 0.1% glutaraldehyde. Reactions were stopped after 2 and 10 min by addition of 1 M Tris pH 7.5 and crosslinking products were analyzed by SDS-PAGE (4–15%) followed by Sypro Ruby Staining (ThermoFisher).

### Hydrogen/deuterium exchange coupled to mass spectrometry (HX-MS)

HX-MS experiments were performed as described earlier (Rist et al., 2003). Fifty picomolar of ClpB (wt and derivatives) was incubated for 3 min at 30°C in low salt MDH buffer (50 mM Tris pH 7.5, 20 mM KCl, 20 mM MgCl_2_, 2 mM DTT) in presence of 2 mM ATPγS. Next ClpB was diluted 20-fold into respective D_2_O-based low salt MDH buffer to initiate amide hydrogen exchange. The exchange reaction was quenched by the addition of 1 volume of ice-cold quench buffer (0.4 M potassium phosphate pH 2.2) and injected into an UPLC (Waters) setup, following online peptic digestion. ClpB peptides were analyzed on an electrospray ionization quadrupole time-of-flight mass spectrometer (MaXis UHR qTOF classic, Bruker Daltonics) as described (Rist et al., 2003). Calculation of centroids was conducted manually in an excel sheet based on the following equation after extraction of I_i_ (peak intensity) and m_i_ (m/z) using the Bruker Compass software (Bruker Daltonics):

$$\begin{array}{l} \langle m\rangle =\frac{\sum {\text{I}}_{\text{i}}{\text{m}}_{\text{i}}}{\sum {\text{I}}_{\text{i}}} \end{array}$$

For initial data analysis also an automatic data analysis software was used (HDExaminer, Sierra Analytics). A fully deuterated ClpB sample was generated by incubating D_2_O in the presence of 8 M GdnHCl and analyzed under the same conditions to correct for back-exchange. The relative amount of deuterium atoms incorporated by each peptic fragment was calculated as:

$$\begin{array}{l} \%D=\frac{mass_{t}-mass_{0\%}}{mass_{100\%}-mass_{0\%}}\times 100 \end{array}$$

where mass_*t*_ is the observed average mass of the peptide at time point t, mass_0%_ is the observed average mass of the undeuterated peptide and mass_100%_ is the observed average mass of the fully deuterated peptide.

### Spot tests

*E. coli* cells harboring plasmid-encoded *clpB* alleles were grown in the absence of IPTG overnight at 30°C. Serial dilutions were prepared, spotted on LB-plates containing different IPTG concentrations and incubated for 24 h at indicated temperatures.

### Fluorescence microscopy

*E. coli* Δ*clpB* cells harboring IPTG-inducible YFP-tagged *clpB* alleles were grown to mid-exponential growth phase in the presence of 100 μM IPTG at 30°C. Cells were subjected to heat stress (20 min at 43°C) followed by a recovery period (120 min) at 30°C. One milliliter cell cultures were taken before and after heat stress and during recovery and centrifuged. For snapshot imaging, cells pellets were resuspended in 100 μl icecold PBS buffer and immobilized on 1% (w/v) agarose pads (in 1x PBS). Agarose pads were sealed with Apiezon grease and covered with cover slips. Imaging was performed using the xcellence IX81 wide field system (Olympus) with a Plan Apochromat x100/1.45 numerical aperture oil objective, a Hamamatsu OrcaR2 camera and the according filter settings (YFP). For image analysis ImageJ was used and for statistical analysis at least 100 cells were counted to determine the % of cells without and with foci pre, after heat shock and during the recovery phase.

## Results

### Mutating the interface residue A328 affects cellular toxicity of ClpB wild type and hyperactive K476C

**Figure 1:**
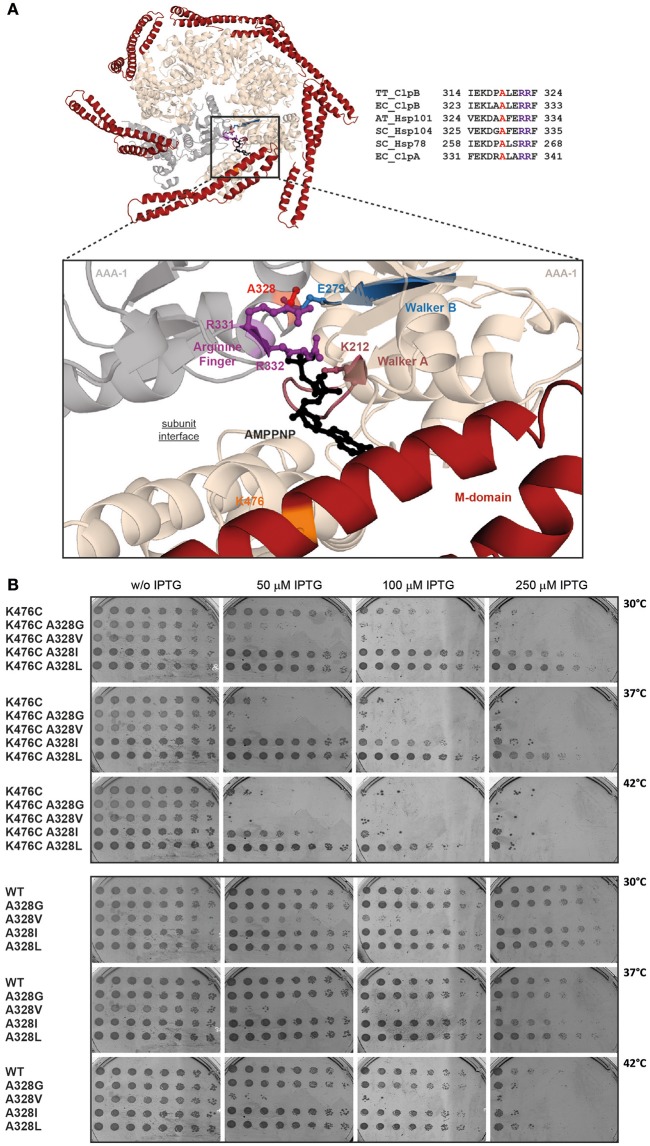
**The conserved intersubunit residue A328 controls ClpB activity. (A)** Hexameric model of AAA-1 ring of *E. coli* ClpB. Structure of the hexameric AAA-1 ring of *E. coli* ClpB and M-domains (red). The hexameric model is based on the crystal structure of *Thermus thermophilus* ClpB (pdb number 1qvr1) and was generated as described in Diemand and Lupas (2006). The enlarged section shows the catalytic site and bound AMPPNP. AAA-1 subunits are in beige and gray. The positions of Walker A and B motifs, the trans-acting arginine fingers and the analyzed mutational sites (A328, K476) are indicated. AMPPNP is shown in black. A sequence alignment of the analyzed subunit interface of ClpB homologs (Hsp101, Hsp104, Hsp78) and ClpA is provided. A328 is highlighted in red, arginine fingers in purple (TT, *Thermus thermophilus*; EC, *Escherichia coli*; AT, *Arabidopsis thaliana*; SC, *Saccharomyces cerevisiae*). **(B)** *E. coli* Δ*clpB* cells expressing the indicated plasmid-encoded *clpB* alleles under control of an IPTG-regulatable promoter were grown overnight at 30°C. Various dilutions (10^0^–10^−7^) were spotted on LB plates containing the indicated IPTG concentrations and incubated at 30, 37, or 42°C for 24 h.

We set out to study intersubunit communication within the ClpB AAA-1 ring and its connection to subunit coupling of hyperactive ClpB M-domain mutants, exhibiting high ATPase activities. Mutating the classical Arginine-finger of the ClpB AAA-1 domain leads to drastic phenotypes, including oligomerization defects and entire loss of disaggregation activity, thereby affecting further analysis of the coupling mechanism. We therefore aimed at analyzing nearby, conserved residues located at the subunit interface. We made use of a previous genetic study in *Arabidopsis thaliana*, analyzing the plant homolog of ClpB, Hsp101. The authors isolated the Hsp101-A499T mutant, which harbors a point mutation in M-domain helix3 and exhibits cellular toxicity at increased temperatures (38°C), a phenotype not observed for *hsp101* null mutants (Lee et al., 2005). The position of the mutation (M-domain helix 3) and the determined gain-of-function phenotype (toxicity) suggest that Hsp101-A499T represents a hyperactive M-domain mutant. Notably, Hsp101-A499T toxicity could be suppressed by the additional mutation A329V, located at the subunit interface of the AAA-1 ring (Lee et al., 2005). The molecular basis of this suppression activity remained unclear, as a biochemical analysis of Hsp101 wild type and mutants was not performed. The suppressor mutation A329V is located far away from the M-domain mutated site (37 Å based on ClpB structure; Figure 1A). This strongly suggests that the suppressor does not act in an allele-specific manner and does not directly affect M-domain conformation but buffers against a general deregulation of Hsp101 activity caused by M-domain mutation. Here, we used this original genetic information as basis to explore the interplay of ClpB ATPase regulation by M-domains and intersubunit communications. We used ClpB-K476C as hyperactive M-domain variant as it still allows for Hsp70 cooperation (Oguchi et al., 2012). To affect intersubunit communication we mutated the highly conserved A328 residue, which corresponds to Hsp101 A329 isolated as suppressor site of the toxic Hsp101-A499T M-domain mutant (Figure 1A). The residue A328 is located at the subunit interface in close proximity to the arginine fingers R331/R332 of AAA-1, implying a potential role in ClpB ATPase control (Figure 1A).

To test for a role of A328 in regulatory ATPase circuits of *E. coli* ClpB, we changed the size of the residue at the 328 position, creating A328G, A328V, A328L, and A328I variants. These variants were additionally linked to K476C to analyze for suppressing effects toward the hyperactive M-domain mutation. We started our analysis by testing all ClpB variants for temperature-dependent toxicity (Figure 1B). This screen was performed in *E. coli* Δ*clpB* cells expressing respective plasmid-encoded *clpB* alleles from an IPTG-regulatable promoter. Overexpression of ClpB-K476C caused cell death in presence of 250 μM IPTG at 30°C and 50 μM IPTG at 37/42°C. The additional presence of the A328L and A328I mutations either entirely suppressed K476C toxicity (A328L), or strongly reduced toxicity (A328I). Toxicity upon expression of A328I/K476C was only observed at 42°C in presence of 100 μM IPTG (Figure 1B). In contrast, combining A328G or A328V with K476C did not suppress but rather increased toxicity as cell death was already noticed at 30°C in presence of 50 μM IPTG. As control we determined toxicity of single A328 alterations upon expression in *E. coli* Δ*clpB* cells (Figure 1B). Production of ClpB-A328G, A328L, and A328I did not affect cell growth as expected. Surprisingly, expression of *clpB-A328V* was lethal and cellular toxicity was even higher as compared to expression of *clpB-K476C*. The observation that ClpB-A328V is toxic on its own can explain the noticed increased toxicity of ClpB-A328V/K476C. Summing up, the interface residue A328 is highly sensitive to mutational alteration causing either cellular toxicity or toxicity suppression of the otherwise lethal ClpB-K476C M-domain mutant. These findings indicate that A328 plays a crucial role in controlling ClpB activity.

### A328 is crucial for ClpB disaggregation activity

**Figure 2:**
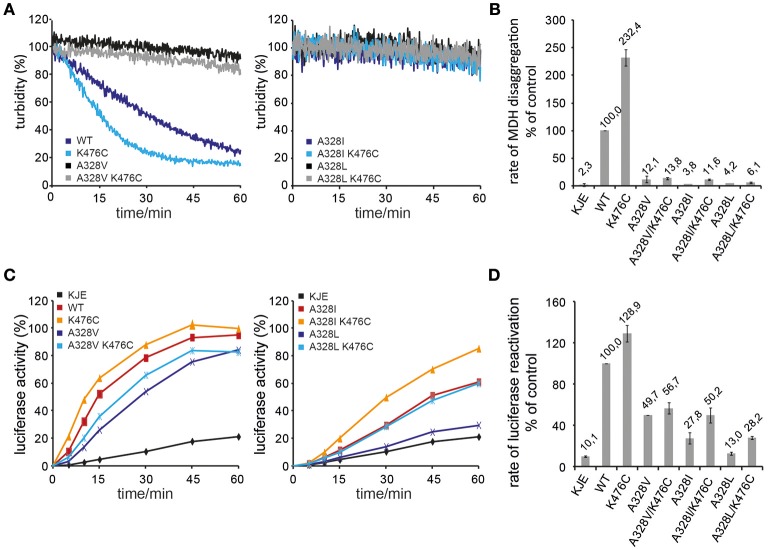
**A328 is crucial for ClpB disaggregation activity**. **(A,B)** Solubilization of aggregated Malate Dehydrogenase (MDH) was monitored by turbidity measurements in the presence of the cooperating *E. coli* DnaK chaperone machinery (KJE), ClpB wild type (WT), and the indicated ClpB mutants. The activity of ClpB WT (MDH disaggregation rate) was set at 100%. A KJE-only control is given. **(C,D)** Refolding of urea-denatured Luciferase was determined in the presence of the cooperating *E. coli* DnaK chaperone machinery (KJE), ClpB wild type (WT), and the indicated ClpB mutants. The activity of ClpB WT (Luciferase refolding rate) was set at 100%. A KJE-only control is given.

We determined the consequences of A328 mutations on ClpB disaggregation activities by using aggregates of heat-denatured Malate Dehydrogenase (MDH) and urea-denatured Luciferase as model substrates. We focused our analysis on A328L, A328I, and A328V variants as those mutants either suppressed K476C toxicity (A328L, A328I) or exhibited toxicity on its own (A328V) (Figure 1B). Disaggregation was performed in presence of the cooperating DnaK (Hsp70) chaperone system (DnaK/DnaJ/GrpE: KJE) as neither ClpB wild type (wt) nor the mutant proteins showed disaggregation activity in absence of the Hsp70 partner (data not shown). Solubilization of MDH aggregates was monitored by determining the decrease in sample turbidity. MDH disaggregation by KJE and ClpB wild type was completed after 60 min. Solubilization of aggregated MDH by hyperactive ClpB-K476C and KJE was completed already after 30 min and the MDH disaggregation rate increased by 2,3 times as compared to ClpB wt (Figures 2A,B). All A328X variants, either alone or in combination with K476C, showed strongly reduced disaggregation activity (Figures 2A,B). ClpB-A328V had 12% disaggregation activity as compared to ClpB wt and this low activity was hardly increased for ClpB-A328V/K476C, indicating a dominant effect of the A328V mutation. A328L mutants (wt or K476C-linked) only showed background activity as determined in presence of KJE only. Low disaggregation activity (11%) was observed for A328I, but only if combined with hyperactive K476C, indicating that the activating K476C mutation can partially restore disaggregation activity of ClpB-A328I (Figures 2A,B).

A similar trend was observed when using aggregates of urea-denatured Luciferase as alternative substrate (Figures 2C,D). The disaggregation and refolding of urea-denatured Luciferase is slightly less sensitive toward alterations of ClpB activity. We assume that this is caused by differences in the nature of MDH and Luciferase aggregates including size and structure. Extraction of Luciferase molecules from Luciferase aggregates likely requires lower force application as can be also seen by partial activity of KJE in absence of ClpB (10% Luciferase refolding rate by KJE only as compared to KJE/ClpB wt). Luciferase refolding was fastest by KJE/ClpB-K476C, confirming its hyperactive state. All A328X variants showed reduced Luciferase refolding activity to variable degrees (Figures 2C,D). While ClpB-A328L activity was only slightly above the KJE control (13% activity), disaggregation activities of A328I (28%) and A328V (50%) were higher. Linking A328X mutations to hyperactive K476C generally increased disaggregation activities. Increase was largest for A328L/K476C and A328I/K476C and resulted in 28% (A328L/K476C) and 50% (A328I/K476C) Luciferase reactivation activities as compared to ClpB wt (Figures 2C,D). Only a minor increase was noticed for A328V/K476C, exhibiting 57% activity as compared to 50% disaggregation activity determined for ClpB-A328V, resembling results from the MDH disaggregation assays.

Taken together, the disaggregation activities provide a rationale for suppression of ClpB-K476C toxicity by A328L and A328I mutations, as they strongly reduce disaggregation activities and abrogate the high disaggregation activity of K476C. While ClpB-A328V represented the most potent A328X mutant in protein disaggregation, its activity was reduced and clearly different from hyperactive ClpB-K476C. The molecular basis for cellular toxicity noticed upon *clpB-A328V* expression in *E. coli* might therefore be different from ClpB-K476C.

### A328 controls ClpB ATPase activity

**Figure 3:**
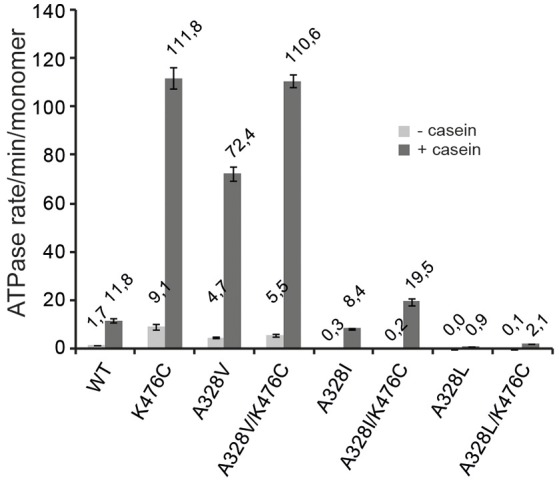
**A328 controls ClpB ATPase activity**. ATPase activities of ClpB wild type (WT) and indicated ClpB mutants were determined in absence (−) and presence (+) of substrate casein.

The hyperactive state of ClpB and Hsp104 M-domain mutants is linked to very high ATP hydrolysis rates in presence of substrate (Oguchi et al., 2012; Lipinska et al., 2013; Kummer et al., 2016). In order to link the determined cellular toxicities and disaggregation activities of ClpB A328X variants to potential changes in ATPase activities, we determined ATP turnover rates in absence (basal rate) and presence (stimulated rate) of the model substrate casein and compared those to ClpB wild type and ClpB-K476C (Figure 3). A328L and A328I mutants (alone or linked to K476C) showed strongly reduced basal ATPase activities. Addition of casein increased ATP turnover by A328I and A328I/K476C, resulting in ATPase activities that were either 3-fold lower (A328I) or 1,65-fold higher (A328I/K476C) as compared to ClpB wild type. In case of the A328L mutation a significant ATPase activity was only determined for A328L/K476C upon addition of casein, however, ATP turnover was still 5,6-fold lower as compared to ClpB wt (+ casein). The determined reductions in ATPase activities of ClpB-A328L and -A328I mutants are overall in agreement with their lowered disaggregation activities and also explain why A328L and A328I suppress K476C toxicity as A328L/K476C and A328I/K476C do not reach the high ATPase activity of K476C. To exclude that the low ATPase activities determined for ClpB-A328I and ClpB-A328L are caused by oligomerization defects, we performed glutaraldehyde crosslinking experiments (Supplementary Figure 1), demonstrating that all investigated ClpB mutants can oligomerize as ClpB wt.

Notably, a very high ATPase rate was determined for ClpB-A328V in presence of casein and ATP turnover was 6,1-fold increased as compared to ClpB wt (Figure 3). This high ATPase activity is reminiscent of hyperactive ClpB-K476C. Combining both ATPase activating mutations in ClpB-A328V/K476C did not result in further ATPase activity increase, presumably because the ATPase motor is already running at maximal speed.

ClpB-A328V exhibits opposing consequences on ATPase activity as compared to the A328L and A328I mutants. These differences in ATPase activities correlate well with the noticed effects of respective mutants on cellular toxicity. The high ATPase activity of ClpB-A328V is linked to cellular toxicity, which is not observed for slowly hydrolyzing ClpB-A328L and ClpB-A328I and respective K476C-linked mutants. The low ATPase activities of ClpB-A328I/L mutants are similar to those determined for arginine finger mutants (Mogk et al., 2003; Yamasaki et al., 2011; Biter et al., 2012). In contrast, the high ATPase activity of A328V is entirely unexpected and therefore we focused further analysis on this particular variant.

### ClpB-A328V is hyperactive and unfolds stable protein domains

**Figure 4:**
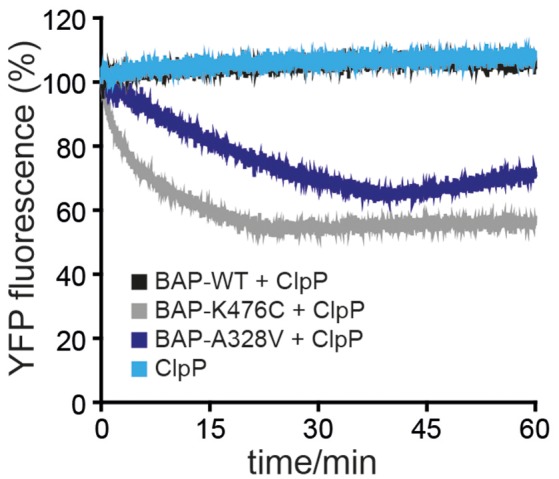
**ClpB-A328V has high unfolding power**. Unfolding and degradation of Casein-YFP by BAP wild type (WT) and mutants in presence of ClpP was followed by monitoring YFP fluorescence.

ClpB-A328V exhibits key characteristics of hyperactive ClpB mutants: (i) very high ATPase activity in presence of substrate (Figure 3) and (ii) cellular toxicity upon expression in *E. coli* cells (Figure 1B). High ATPase rates of hyperactive ClpB M-domain mutants also enable them to unfold stable protein domains, an activity not observed for ClpB wild type (Haslberger et al., 2008; Oguchi et al., 2012). To test for high unfolding activity we made use of casein-YFP, which is recognized by ClpB as substrate via its casein moiety. High unfolding activity of ClpB will allow for YFP unfolding and can be monitored by loss of YFP fluorescence. However, YFP can rapidly refold upon initial unfolding making it difficult to robustly study ClpB unfolding activity. To overcome this obstacle we made use of the ClpB variant BAP, which binds to the *E. coli* peptidase ClpP, thereby directly linking successful substrate unfolding and threading to degradation via associated ClpP (Weibezahn et al., 2004). BAP/ClpP allows determining unfolding activities toward casein-YFP, as unfolding of the YFP moiety results in its degradation and thereby an irreversible loss of YFP fluorescence. Fluorescence of casein-YFP remained stable upon incubation with ClpP or BAP-wt/ClpP, confirming that YFP resists threading by BAP-wt (Figure 4). In contrast, BAP-K476C/ClpP caused rapid loss of YFP fluorescence, decreasing fluorescence intensity to 50% within 20 min. Degradation of Casein-YFP by BAP-K476C/ClpP was not complete. We assume this is caused by heterogeneity of the substrate pool, which includes a fraction that is not accessible for BAP-K476C processing. BAP-A328V/ClpP also degraded Casein-YFP to a similar degree, yet the degradation rates were 6-fold lower as compared to BAP-K476C/ClpP (Figure 4). Still, loss of YFP fluorescence shows that BAP-A328V unfolds the stable YFP moiety in contrast to BAP wt, demonstrating hyperactivity. The A328V mutation therefore fulfills all key characteristics of hyperactive ClpB mutants: (i) cellular toxicity, (ii) high ATPase activity, and (iii) high unfolding activity.

### Low disaggregation activity of A328V is not caused by alterations in protein aggregate targeting

**Figure 5:**
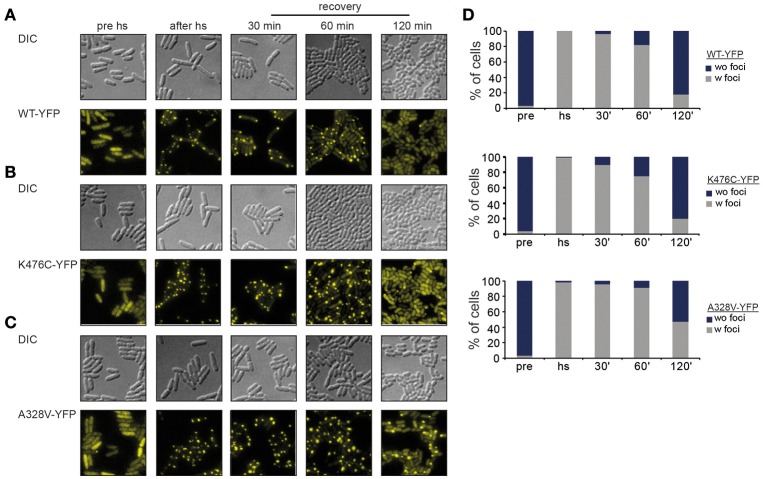
**ClpB-A328V is not affected in protein aggregate targeting. (A–C)** *E. coli* Δ*clpB* cells expressing the indicated plasmid-encoded *clpB-yfp* alleles under control of an IPTG-regulatable promoter were grown to mid-exponential growth phase at 30°C, heat shocked to 43°C for 20 min and shifted back to 30°C. The cellular localization of ClpB-YFP (WT and derivatives) was monitored by fluorescence microscopy before heat shock (pre hs), directly after heat shock (after hs) and at the indicated time points during the recovery period at 30°C. **(D)** Binding of ClpB-YFP WT and derivatives to polar protein aggregates formed at 43°C. Numbers of cells (%) showing stress-induced fluorescent ClpB-YFP foci (w foci) or only diffuse fluorescence (wo foci) were determined (*n* > 100).

ClpB-A328V and ClpB-K476C share key characteristics of an hyperactive activity status but differ substantially in disaggregation activities *in vitro*. While ClpB-K476C exhibits superior disaggregation activity, ClpB-A328V activity is low. We speculated that this difference might stem from reduced binding of ClpB-A328V to the DnaK partner chaperone, resulting in less efficient targeting to protein aggregates. We employed fluorescence microscopy using C-terminal YFP fusions to ClpB-wt or -A328V and -K476C mutants to monitor their DnaK-dependent binding to protein aggregates. To avoid cellular toxicity upon overexpression of hyperactive *clpB-K476C* and *clpB-A328V* mutants all *yfp*-fused *clpB* constructs were expressed in *E. coli* Δ*clpB* cells from a low copy vector to produce approx. ClpB wt levels (data not shown). These low expression levels do not affect growth of *E. coli* cells. Protein aggregates forming in *E. coli* cells during heat stress are deposited at the cell poles (Winkler et al., 2010). ClpB-YFP is recruited to polar protein aggregates in a DnaK-dependent manner (Winkler et al., 2012) leading to the appearance of polar ClpB-YFP foci after heat stress (45°C) at the expense of diffuse cytosolic ClpB-YFP fluorescence (Figure 5A). Polar ClpB-YFP fluorescence vanished during a recovery period at 30°C within 120 min in >80% of cells. Loss of polar ClpB-YFP fluorescence was accompanied with the reappearance of diffuse cytosolic ClpB-YFP fluorescence, reflecting successful protein disaggregation (Figures 5A,D). We next monitored the cellular distribution of ClpB-K476C-YFP and ClpB-A328V-YFP during stress application. Both constructs exhibited diffuse fluorescence before heat shock but formed polar foci upon heat shock in a manner indistinguishable from ClpB-wt-YFP (Figures 5B,C). These findings exclude that defects in DnaK interaction are causative for reduced ClpB-A328V disaggregation activity. Notably, loss of ClpB-A328V-YFP foci during the recovery period was delayed and half of the cell population still contained polar foci after 120 min (Figure 5D). This is indicative of a reduced disaggregation activity of ClpB-A328V-YFP *in vivo*, in agreement with results obtained for aggregated MDH and Luciferase model substrates *in vitro*. We conclude that ClpB-A328V is affected in a step of the disaggregation cycle downstream of DnaK-mediated targeting to protein aggregates.

### ClpB-A328V and ClpB-K476C differ in the mechanistic basis of ATPase hyperactivity

**Figure 6:**
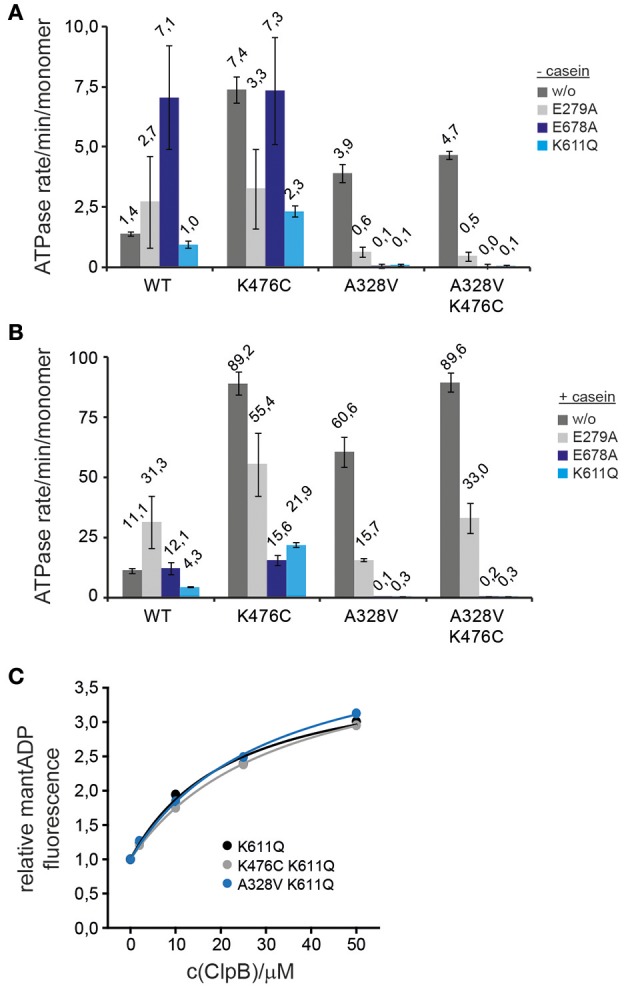
**ClpB-A328V stimulates ATP turnover at AAA-2. (A,B)** ATPase activities of ClpB wild type (WT) and indicated ClpB mutants were determined in absence (−) and presence (+) of substrate casein. Activities of ClpB WT and A328V, K476C, A328V/K476C mutants were analyzed either alone (w/o) or when additionally linked to Walker B (E279A, E678A) and Walker A (K611Q) mutants. **(C)** Binding of indicated ClpB Walker A mutants (K611Q and indicated derivatives) to mantADP. The fluorescence intensity of mantADP in absence of ClpB proteins was set at 1.

The absence of an obvious defect of ClpB-A328V in DnaK interaction let us to speculate that the molecular basis of ClpB-A328V and ClpB-K476C hyperactivity—reflected by high ATP turnover rates—differs between the two mutants. To analyze for differing effects of ClpB-A328V and ClpB-K476C on the ATPase cycle, we linked the mutations to single ClpB Walker B mutations in AAA-1 (E279A) or AAA-2 (E678A) allowing for ATP binding but abolishing ATP turnover in the respective AAA+ ring. Additionally, we combined the A328V and K476C mutations with the Walker A mutation K611Q, causing deficiency in ATP binding at the AAA-2 ring. We did not include a respective Walker A mutant of AAA-1 as it shows oligomerization defects (Watanabe et al., 2002; Mogk et al., 2003). We determined ATPase activities in absence and presence of substrate casein and included single ClpB Walker B and A mutants as reference (Figures 6A,B).

The effects of the tested ClpB mutants on ATPase activities were complex. Linking K476C to E279A or K611Q reduced but did not abolish high ATPase activity in presence of casein (Figures 6A,B). ClpB-K476C/E678A did not exhibit increased ATPase activity as compared to ClpB-E678A and ATP turnover was no longer stimulated by casein. We conclude that K476C leads to increased ATP turnover in both AAA rings, however, freezing the AAA-2 ring in the ATP state (E678A) almost entirely prevents casein-dependent ATPase stimulation by the K476C M-domain mutation. The latter effect is also observed for ClpB-E678A, indicating that substrate binding predominantly stimulates ATP turnover at AAA-2. Furthermore, reductions in ATPase activities were most pronounced when linking K476C to Walker A/B mutants of the AAA-2 ring, suggesting that the increased ATP turnover in the hyperactive M-domain mutant K476C is mostly due to increased ATPase activity in the AAA-2 ring.

The results obtained for A328V linked to Walker A and B mutations were different from K476C. The ClpB-E279A/A328V double mutant exhibited reduced ATPase activity compared to respective single mutants, suggesting that each mutation affects the ClpB ATPase cycle differently, leading to a distinct effect upon combining both mutations. Linking A328V to Walker A or B mutants of AAA-2 entirely abrogated basal and casein-stimulated ATPase activity, in contrast to ClpB-K476C (Figures 6A,B). This indicates that ATP turnover at AAA-2 is obligatory for ClpB-A328V ATPase activity and suggests that ATP binding or turnover at AAA-1 might be abrogated in ClpB-A328V. The ClpB-A328V-K476C mutant also did not exhibit ATPase activity when linked to K611Q or E678A (Figures 6A,B), demonstrating that the effect of A328V on ATPase control is dominating and cannot be compensated by M-domain mediated ATPase regulation.

The absence of ClpB-A328V ATPase activity if linked to Walker A and B mutants of AAA-2 could be explained by A328V abrogating nucleotide binding at AAA-1. To test for potential nucleotide binding defects we used the fluorescent nucleotide analog mantADP, which shows increased and blue-shifted fluorescence upon binding to ClpB (Schlee et al., 2001). Binding of mantADP was largely unaltered for ClpB-A328V as compared to ClpB wt or ClpB-K476C, and only a 2,2-fold decrease in affinity was determined (K_D_: 0,51 μM for ClpB-A328V vs. 0,23 and 0,25 μM for ClpB wt and ClpB-K476C; Supplementary Figure 2A). Similarly, competition titration experiments with ADP or ATPγS did not reveal strong differences in nucleotide binding as respective *K*_D_-values of A328V were again only 2-fold increased as compared to ClpB wt (Supplementary Figure 2B). To specifically test for mantADP binding at AAA-1 only we analyzed ClpB-A328V/K611Q, which is deficient in nucleotide binding at AAA-2. mantADP binding curves of ClpB wt and ClpB-A328V were indistinguishable (Figure 6C). We were not able to determine *K*_D_-values as we did not reach binding saturation in presence of 50 μM ClpB protein. This is explained by a low nucleotide binding affinity of AAA-1 if AAA-2 stays nucleotide-free (Fernandez-Higuero et al., 2011). Together these findings exclude nucleotide-binding defects of ClpB-A328V. The lack of any ATPase activity determined for ClpB-A328V/E678A and ClpB-A328V/K611A therefore implies that (i) ClpB-A328V is deficient in ATP turnover at AAA-1 and (ii) the strongly increased ATPase activity of ClpB-A328V in presence of casein is caused by exclusively stimulating ATP turnover at AAA-2. Conversely, preventing nucleotide binding or hydrolysis at AAA-2 might affect the ATPase cycle of ClpB-A328V in a more complex manner, abrogating ATP hydrolysis in the entire ClpB hexamer once AAA-2 activity is blocked. The noticed differences in ATPase activities of A328V and K476C mutants when linked to Walker A or B mutants also demonstrate that the molecular basis for their hyperactive activity states must be different. ClpB-A328V and ClpB-K476C therefore represent different classes of hyperactive ClpB mutants.

### ClpB-A328V affects the conformation of the Walker a motif of AAA-2

**Figure 7:**
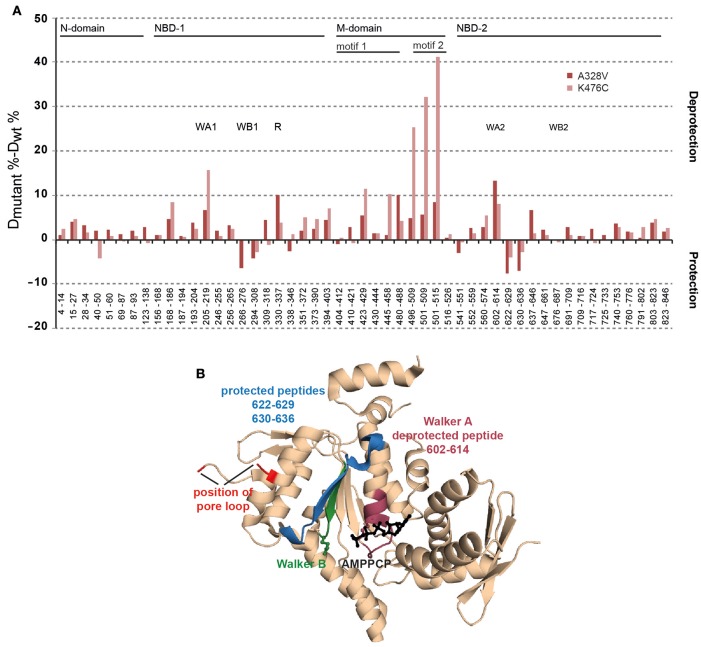
**ClpB-A328V shows specific conformational changes in peptic peptides including key elements of AAA domains. (A)** Changes in structural dynamics of ClpB-A328V. Difference in deuteron incorporation (HX) after 60 s between either ClpB-A328V or ClpB-K476C and ClpB-WT in the ATPγS-bound state. A ClpB domain organization is given. Peptic peptides including Walker A and B motifs (WA, WB) or arginine fingers (R) are indicated. **(B)** Structure of the *T. thermophilus* AAA-2 domain in the AMPPCP-bound state (pdb-number 4lj6). Corresponding *E. coli* ClpB-A328V peptides located at the catalytic site showing decreased (purple) or increased (blue) protection in HX as compared to ClpB WT are indicated. The position of the Walker B motif (green) and the substrate threading pore loop (red) is indicated. AMPPCP is shown in black.

The determined effects of hyperactive ClpB-A328V on ATPase and unfolding activities must stem from specific conformational changes within the ClpB ring. To study for potential effects of the A328V mutation on AAA-1, AAA-2, and M-domain conformations we determined the structural flexibility of ClpB-A328V by amide hydrogen exchange (HX) mass spectrometry (MS). HX-MS determines the solvent accessibility and structural flexibility of the peptide backbone. Amide hydrogens are protected from HX if engaged through hydrogen bonds in secondary and tertiary structures. We compared the HX-MS patterns of peptic peptides from ClpB wt, ClpB-A328V, and ClpB-K476C in the presence of ATPγS (Figure 7A). The HX-MS pattern of ClpB-A328V was overall similar to ClpB wt and differences in HX were lower than 5% for most peptic peptides. This confirms nucleotide binding to both AAA domains of ClpB-A328V, as a defect in nucleotide binding to either AAA domain would result in strong deprotection of multiple peptic peptides (Oguchi et al., 2012). ClpB-A328V did not exhibit strong deprotection of M-domain motif2 peptic peptides as observed for ClpB-K476C (Figure 7A). ClpB-A328V hyperactivity therefore does not rely on dissociation of M-domain motif2, confirming that the molecular basis of ClpB-K476C and ClpB-A328V hyperactivities is different. Further analysis revealed that only two out of the multiple AAA-1 and AAA-2 peptic peptides of ClpB-A328V showed a deviation of 10% or more in HX as compared to ClpB wt. The first peptide E330-F337 includes the arginine fingers R331 and R332 of AAA-1 and exhibits a 10% increase in HX compared to ClpB wt. This suggests an altered positioning of the arginine fingers in ClpB-A328V. The second peptide L602-L614 (13% increase in HX) is encompassing the Walker A motif of AAA-2 (G605-T612). Notably changes in HX were also observed for other ClpB-A328V peptic peptides of AAA-2 located close to the Walker A peptide (Figures 7A,B). This implies structural differences in the catalytic ATPase center of the AAA-2 domains of ClpB wt and ClpB-A328V. An increased deprotection of the AAA-2 Walker A peptic peptide L602-T612, though not as pronounced (8% increased HX) was also noticed for ClpB-K476C. ClpB-K476C also showed strong deprotection of I205-L219 (16% change in HX), encompassing the Walker A motif of AAA-1 (G206-T212). Here, ClpB-A328V also showed increased deprotection, yet not to the same degree. Therefore, both hyperactive mutants, ClpB-A328V and ClpB-K476C, exhibit specific structural changes in the catalytic centers that were most pronounced either in AAA-1 (K476C) or in AAA-2 (A328V), providing a structural correlative to ATPase hyperactivity.

## Discussion

In the presented work we analyzed the role of intersubunit communication in controlling ClpB ATPase and disaggregation activity. We selected the conserved A328 residue for analysis as it is located in a strategic position at the AAA-1 subunit interfaces close to the essential arginine fingers R331 and R332. Furthermore, the identical residue was identified as intragenic suppressor of a toxic, gain-of-function Hsp101 M-domain mutant (Lee et al., 2005), indicating a role of this residue in controlling ClpB/Hsp101 activity. This observation also provided rationale for analysis of a potential interconnection of ClpB intersubunit communication via A328 and M-domain mediated ATPase control.

Our analysis confirms and extends previous findings on intersubunit communication based on arginine finger mutants (Mogk et al., 2003; Werbeck et al., 2011; Yamasaki et al., 2011, 2015; Biter et al., 2012; Zeymer et al., 2014b). We show that A328, like the arginine fingers, acts in both, intra-ring and inter-ring signaling. The alanine residue is conserved in the AAA-1 domain of Class I Hsp100 proteins (e.g., ClpA, ClpB, ClpC, ClpE, ClpV) harboring two AAA modules, but can also be found in other AAA+ family members including CDC48 and NSF (Supplementary Figure 4). This suggests that the regulatory function of A328 uncovered here for ClpB is widespread and operative in various AAA+ proteins. A328X mutants do not only impact on the ATPase activity of the AAA-1 cis ring, but also that of the AAA-2 trans ring. The residue A328 exhibits remarkable sensitivity toward the introduced mutations, justifying its high degree of conservation. While all characterized A328X mutants exhibited reduced disaggregation activities to varying degrees, they showed diverging consequences on ATP hydrolysis rates. A328I and A328L had reduced or almost no ATPase activity, whereas A328V hydrolyzed ATP 6-times faster than ClpB wt in presence of substrate casein. This shows that small structural alterations, caused by the additional presence of a single methyl group in A328L/I compared to A328V, have diverse effects on ATPase activity control in the ClpB hexamer. We suggest that the A328X mutations affect the orientation of the nearby arginine fingers. This is supported for ClpB-A328V by HX-MS analysis, revealing a specific conformational change of the AAA-1 peptic peptide E330-F337 encompassing the arginine fingers R331/R332 (Figure 7A). We assume that subtle differences in arginine finger conformations between A328I/L and A328V variants are basis for their dramatically different ATPase activities.

While the low ATPase activities of A328L/I were expected, the high ATPase rate of ClpB-A328V is surprising. Further analysis revealed that A328V represents a hyperactive ClpB mutant, which shares key characteristics with hyperactive ClpB M-domain mutants (e.g. K476C): high ATPase and unfolding activities and temperature-dependent cellular toxicity. ClpB-A328V, however, differs from M-domain mutants and constitutes a novel class of hyperactive ClpB mutants. HX-MS analysis revealed that M-domain motif2 of ClpB-A328V does not show increased deprotection as compared to ClpB wt, indicating that M-domain motif2 is not displaced from the AAA-1 ring in contrast to ClpB-K476C. This shows that the initial molecular event resulting in high ATPase rates of ClpB-A328V and ClpB-K476C is different. A328V might, however, activate an allosteric network, which is also involved in ATPase activation upon M-domain dissociation. The latter event must include additional activation steps as both classes of hyperactive ClpB mutants (A328V and K476C) differ in disaggregation activities and ATPase regulation. Accordingly, coupling A328V or K476C to Walker B mutants that prevent ATP hydrolysis at the AAA-1 or AAA-2 ring, had distinct consequences on ATPase activities. Preventing nucleotide binding or hydrolysis at AAA-2 entirely abrogated ATPase activity of ClpB-A328V, in contrast to ClpB-K476C (Figure 6). These findings imply that ATP hydrolysis in the AAA-1 ring is blocked in ClpB-A328V. This defect likely affects cooperation of ClpB-A328V with its Hsp70 partner DnaK. While aggregate targeting of ClpB-A328V by DnaK is unaffected (Figures 5C,D), it exhibits low disaggregation activity indicating that ClpB-A328V/DnaK cooperation is affected post recruitment. We suggest that efficient substrate transfer from DnaK to ClpB requires ATP turnover at the AAA-1 ring, an activity not performed by ClpB-A328V. This is explaining why ClpB-A328V has reduced disaggregation activity, whereas ClpB-K476C is highly potent *in vitro*.

However, ClpB-A328V is different from the ClpB Walker B mutant E279A, which also blocks ATP hydrolysis in the AAA-1 ring. In contrast to ClpB-A328V (which has increased ATPase activity through AAA-2) ClpB-E279A has lower ATPase activity (−/+ casein; Figure 6) and hardly exhibits cellular toxicity (Supplementary Figure 3). Also, combining A328V and E279A strongly reduced ATPase activity and cellular toxicity (Supplementary Figure 3). The deregulation of ATPase control in AAA-2 caused by either A328V or E279A mutations is therefore different. This difference might be explained by defects of A328V in sensing the nucleotide state (ATP) and transmitting this signal within the cis AAA-1 and to the trans AAA-2 ring. Loss of ATPase regulation involving A328V causes strongly increased ATP turnover at AAA-2 (Figure 6). The AAA-2 ring is providing the main threading power and an increase in its ATPase activity explains the high unfolding power of ClpB-A328V and likely its cellular toxicity. HX-MS analysis of ClpB-A328V revealed specific conformational changes in the catalytic center of AAA-2. The peptic peptide L602-T612, including the Walker A motif of AAA-2, showed increased HX, indicating increased structural flexibility at the catalytic site. Notably, determination and analysis of ClpB AAA-2 crystal structures revealed that the catalytic site of AAA-2 is inactive as the essential Walker A lysine residue (K611) exists in a stretched conformation and does not contact bound nucleotide (Zeymer et al., 2014a). It is tempting to speculate that the increased flexibility determined for the AAA-2 Walker A peptide of ClpB-A328V reflects a repositioning of K611 and therefore activation of the AAA-2 ATPase motor.

Linking A328X mutations to hyperactive ClpB-K476C also offers an explanation for the original identification of the intersubunit residue as suppressor of a toxic Hsp101 M-domain mutant (Lee et al., 2005). ATPase and disaggregation activities of ClpB-A328I/L-K476C double mutants are low. As cellular toxicities of ClpB M-domain mutants correlate with high ATPase activities, the determined reduction in ATP turnover explains the suppressor function of the A328I/L mutation. Linking the K476C mutation to A328I/L still increases ATPase and disaggregation activity. This indicates that the primary signaling routes controlled by the M-domain and A328 are distinct in parts and in the ClpB-A328I/L-K476C double mutant, up-regulation by one pathway (K467C) is counteracted by downregulation of the other pathway (A328I/L).

## Author contributions

Conceived and designed experiments: KBF, BB, AM. Performed experiments: KBF. Analyzed the data: KBF, BB, AM. Wrote the manuscript: BB, AM.

## Funding

This work was funded by grants of the Deutsche Forschungsgemeinschaft (BB617/17-2 and MO 970/4-2) to BB and AM.

### Conflict of interest statement

The authors declare that the research was conducted in the absence of any commercial or financial relationships that could be construed as a potential conflict of interest.
