# Supplementary material for: Mutant Analysis Reveals Allosteric Regulation of ClpB Disaggregase
Source: Front Mol Biosci. 2017 Feb 22;4:6. doi: 10.3389/fmolb.2017.00006 (PMC5319980; doi:10.3389/fmolb.2017.00006)
Supplement: Supplementary file 1 [file Presentation1.PDF]

supp Figure 1

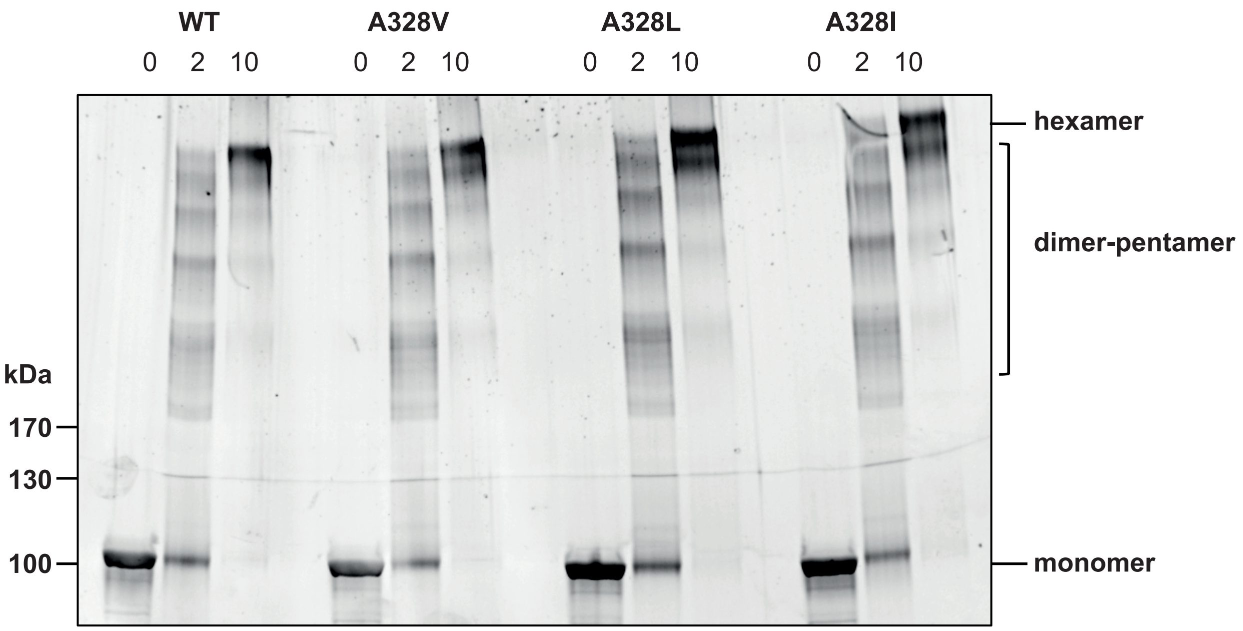

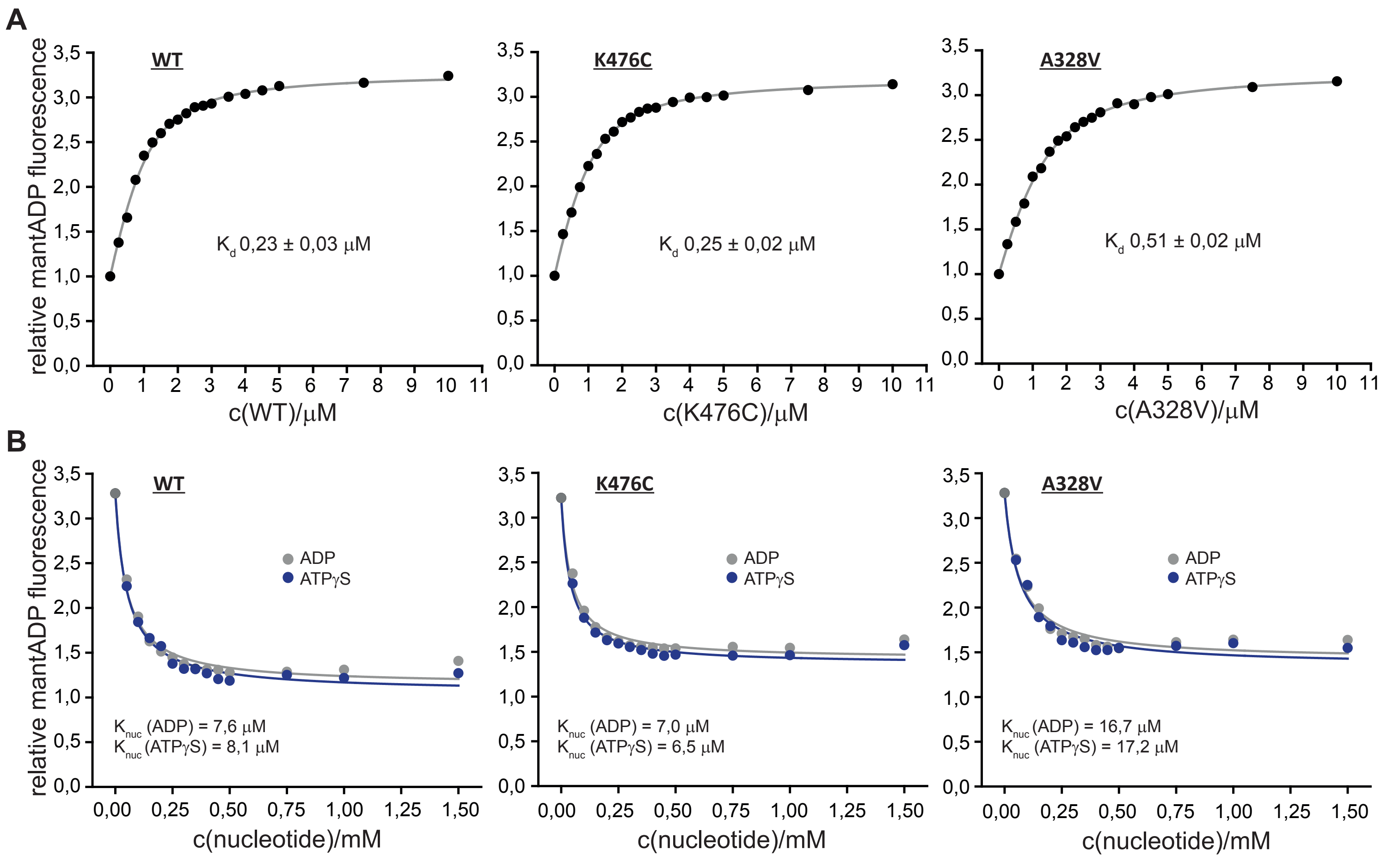

supp Figure 2

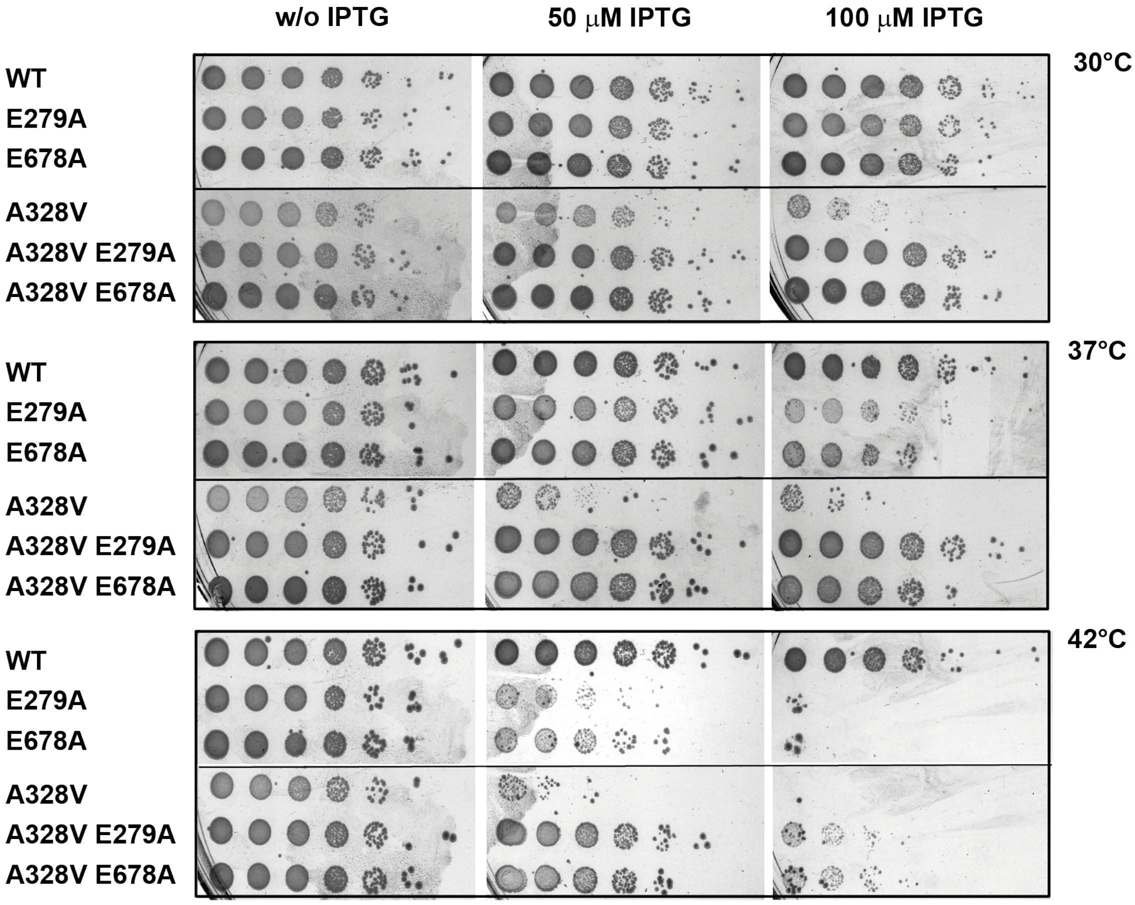

supp Figure 3

A

EC_ClpA	331-FEKDRALARRF-341
EC_ClpB	323-IEKDAALERRF-333
BS_ClpC	324-IEKDAALERRF-334
BS_ClpE	234-IEKDAALERRF-244
VC_ClpV	341-FEKDPALTRRF-351

B

SC_Cdc48-AAA-1	356-ATNRPNSIDPALRRFGRFDREVDIGI-381
SC_Cdc48-AAA-2	632-ATNRPDQIDPAILRPGRLDQLIYVPL-657
CG_NSF-D1	372-MTNRPDLIDEALLRPGRLEVKMEIGL-397
EC_FtsH	296-ATNRPDVLDPALLRPGRFDRQVVVGL-321

supp Figure 4

Supplementary Figure legends

Supplementary Figure 1

Oligomerization of ClpB wild type (WT) and derivatives revealed by crosslinking. ClpB WT and mutants were incubated for 5 min at 25°C in presence of 2 mM ATPγS. Crosslinking reactions were started by addition of glutaraldehyde and proceeded for 2 and 10 min. Crosslinking products were separated by SDS-PAGE, followed by Sypro-Ruby staining.

Supplementary Figure 2

Nucleotide binding properties of ClpB wild type. **(A)**1,5 μM mantADP was incubated with increasing concentrations of ClpB wild type and derivatives and the increase in mantADP fluorescence was determined. The fluorescence intensity of mantADP in absence of ClpB proteins was set at 1. Determined dissociation constants (K_d) are given. **(B)** Increasing nucleotide concentrations (ADP, ATPγS) were added to a preformed ClpB-mantADP complex (1,25 μM MANTADP, 1,25 μM ClpB WT or derivatives). Determined dissociation constants (K_d) are given.

Supplementary Figure 3

Linking hyperactive ClpB-A328V to Walker B mutations reduces cellular toxicity. *E. coli ΔclpB* cells expressing the indicated plasmid-encoded *clpB* alleles under control of an IPTG-regulatable promoter were grown overnight at 30°C. Various dilutions (10^{-1} – 10^{-7}) were spotted on LB plates containing the indicated IPTG concentrations and incubated at 30°C, 37°C or 42°C for 24 h.

Supplementary Figure 4

A328 is conserved in the AAA-1 domain of Class I Hsp100 proteins and other AAA+ proteins. **(A)** Sequence alignment of the analyzed AAA-1 subunit interface of Class I Hsp100 proteins (ClpA, ClpB, ClpC, ClpE, ClpV). A328 is highlighted in red, arginine fingers in purple (EC, *Escherichia coli*; BS, *Bacillus subtilis*; VC, *Vibrio cholerae*). **(B)** Sequence alignment of the AAA subunit interface of selected AAA+ proteins (CDC48, NSF, FtsH). Potential arginine fingers are highlighted in purple, the alanine residue corresponding to A328 of ClpB in red (SC, *Saccharomyces cerevisiae*; CG, *Cricetulus griseus* (Chinese hamster); EC, *E. coli*).
